# Epidemiology of Fungal Periprosthetic Joint Infection: A Systematic Review of the Literature

**DOI:** 10.3390/microorganisms11010084

**Published:** 2022-12-28

**Authors:** Andrea Sambri, Renato Zunarelli, Michele Fiore, Marta Bortoli, Azzurra Paolucci, Matteo Filippini, Eleonora Zamparini, Sara Tedeschi, Pierluigi Viale, Massimiliano De Paolis

**Affiliations:** 1 Orthopaedic Unit, IRCCS Azienda Ospedaliero-Universitaria di Bologna, 40138 Bologna, Italy; 2 Infectious Disease Unit, IRCCS Azienda Ospedaliero-Universitaria di Bologna, 40138 Bologna, Italy

**Keywords:** fungal, prosthetic joint infection, two-stage, one-stage, hip, knee

## Abstract

Fungal prosthetic joint infection (fPJI) is a rare complication; nonetheless, it represents a significant diagnostic and therapeutic challenge. There are no official guidelines on the most effective approach to identify and treat fPJIs. This systematic review aims to review the current literature on fPJI management and provide a comprehensive overview of this topic, especially from an epidemiologic point of view. Studies eligible for this systematic review were identified through an electronic systematic search of PubMed, Scopus, and Web of Science until 30 September 2022. Further references were obtained by cross-referencing. Sixty-three studies met the inclusion criteria, reporting on 372 cases of fPJI; such cases were described mostly in case reports and small case series with only a few larger cohort studies. Diagnosis of fPJI is challenging because of its chronic and indolent clinical course; it is further complicated by the technical difficulty of harvesting fungal cultures. A two-stage revision was the primary procedure in 239 (64.2%) patients whereas DAIR and one-stage approaches were reported in 30 (8.0%) and 18 (4.8 %) cases. In conclusion, our study highlights the heterogeneity of the reported treatments of fPJI, particularly in terms of medical management. With concern to a surgical approach, a two-stage revision arthroplasty is generally suggested, considering fPJI a delayed or late infection. The need for multicenter, prospective studies to provide standardized protocols and improve the treatment of fungal PJI clearly emerges.

## 1. Introduction

Prosthetic joint infection (PJI) is one of the most common complications following arthroplasty, occurring in as many as 0.7% of total hip arthroplasties (THA) and 2% of total knee arthroplasties [1,2]. PJIs are mostly caused by bacteria, especially the *Staphylococcus* species, while fungal PJIs (fPJIs) are much rarer, accounting for about 1% of total PJIs [3,4]. Although several species of fungi may cause PJI, the most common is the *Candida* species [3,5]. In contrast to bacterial PJIs, the clinical presentation of fPJIs tends to be more subtle; acute onset of symptoms is unlikely. Thus, diagnosis is often late, [6] resulting in delayed initiation of appropriate therapy.

Fungi are notoriously difficult to isolate in culture due to the need for specialized culture mediums and longer incubation periods [7]. In some culture-negative cases, uncultivable organisms must be taken into account and alternative identification techniques must be considered [6]. Furthermore, the isolation of a fungus does not exclude the presence of bacteria: concomitant bacterial infection is shown to occur in 15% and 20% of fPJI cases [3]. Bacteria and fungi are in fact thought to act synergistically within the prosthetic biofilm to produce more virulent infections [8,9]. 

Fungal PJI is well known for having a much higher failure rate than bacterial infections [10]. with rates of eradication of infection ranging between 50%+ [11] and 93% [5] in different studies. There are no established guidelines for the treatment of fPJI. Treatment options include antifungal drugs, debridement and implant retention (DAIR), and one- and two-stage revision approaches with variable outcomes [5,12,13,14,15]. Among them, a two-stage approach is widely accepted as the treatment of choice [16,17]. Controversies also exist about the ideal interval between implant removal and reimplantation, the usefulness of antifungal-loaded cement spacers, and the duration of systemic antifungal treatment [18].

The aim of this systematic review is to summarize the current evidence on the diagnosis and management of fungal PJI and to provide a comprehensive overview of this topic, especially from an epidemiologic point of view.

## 2. Material and Methods 

This systematic review was conducted in accordance with the Preferred Reporting Items of Systematic Reviews (PRISMA) guidelines [19,20]. Studies eligible for this systematic review were identified through an electronic systematic search of PubMed, Scopus, and Web of Science until 30 September 2022. The search string used was (spacer OR infection OR prosthesis) AND (mycotic OR fungal).

Resulting articles were screened by the relevance of titles and abstracts; when the abstract provided insufficient information about inclusion and exclusion criteria, the full-text article was examined. 

The following were considered preliminary exclusion criteria: publication in non-peer-reviewed journals, lack of an abstract, and duplicates of already included papers. Animal model studies, biomechanical reports, technical notes, letters to editors, cadaver or in vitro investigations, and instructional were also excluded. Due to the limited amount of relevant literature, study design and location were not considered to further refine the results. No restrictions regarding pathogen type or treatment strategy were employed.

Articles that were considered relevant by the electronic search were retrieved in full-text and a hand-search of their bibliography was performed to find further related articles. Reviews and meta-analyses were also analyzed for cross-referencing purposes. Articles with insufficient details about study populations, surgical intervention, and type of reconstruction were excluded. The remaining studies were categorized by study type according to the Oxford Centre for Evidence-Based Medicine (OCEBM) [21]. All the included studies were reviewed, and data related to topics of interest were extracted and summarized (Table 1 and Table 2).

The success of the treatment was defined as the achievement of infection control at the last follow-up (the absence of clinical and/or radiological and/or laboratory signs of infection, as mentioned in the individual papers). Failure of the treatment was defined as the persistence of infection, reinfection, or no reimplantation; the repetition of surgical debridement in the interstage phase due to the persistence of infection was not considered a failure when it eventually resulted in successful control of the infection at last follow-up after the end of the treatment. 

The study is descriptive, and data are presented as total frequencies and percentages. The heterogeneity and small sample size of most of the included studies did not allow any statistical analysis.

## 3. Results

A total of 63 studies on fungal PJI were found. A total of 372 fungal PJI cases were retrieved, most of which were collected from case reports [12,14,22,23,24,25,26,27,28,29,30,31,32,33,34,35,36,37,38,39,40,41,42,43,44,45,46,47,48,49,50,51,52,53,54,55,56,57,58,59,60,61,62], small case series [15,63,64,65,66,67,68,69,70,71], and only a few from cohort studies with larger samples [3,4,5,8,11,72,73,74,75,76] (Table 1) Two studies reported an incidence of fungal PJI of 1.9% [8] and 12.4% [73] in the whole PJI cohort. 

Among the fungal infections included, 161 were total hip arthroplasties (THA), 207 total knee arthroplasties (TKA), one total elbow arthroplasty, and three total shoulder arthroplasties. Three patients had a bilateral TKA infection [5,76]. The mean age across all studies was 64.8 ± 14.2 years. The mean follow-up period was 27 months, ranging between 3 and 136 months. However, not all the included studies reported on the duration of follow-up.

Only a few studies reported on risk factors for fPJI [3,11,17,18,30,63,74,75,77,78,79]. Despite a high heterogeneity, diabetes, prolonged use of antibiotics, a previous PJI, and immunosuppression were generally recognized as important risk factors for fPJI.

**Table 1:** Review of the Literature.

Study	PJI (Total), n	PJI (Mycotic), n	Site				Age, Years (Mean)	Follow-up, Months (Mean)	Timing of PJI, Months (Mean)	Treatment						No Reimplantation (n)	Medical Treatment Duration	Medical Treatment	PJI Recurrence	Amputations for Recurrence	Timing PJI Recurrence, Months (Mean)
			THA	TKA	SHOULDER	ELBOW				ONE-STAGE	TWO-STAGE	THREE-STAGE	DAIR	Medical	Other						
Theil et al. [4]	26	26	18	8	-	-	72	27	NR	2	24	-	-	-	-	Died: 3 Girdlestone: 3	NR	Caspofungin: 14 Micafungin: 4 Fluconazole: 3 Amphotericin B: 2 Voriconazole: 2 Anidulafungin: 1	4	4	7
Hwang et al. [5]	28	30 *	-	30	-	-	69	52	19.6	-	26	-	4	-	-	-	NR	Amphotericin B: 24 Fluconazione: 4	2	0	NR
Geng et al. [63]	8	8	4	4	-	-	60	55	NR	-	8	-	-	-	-	1	NR	Fluconazole: 5 Voriconazole + fluconazole: 1 Fluconazole + Amphotericin + Caspofungin: 1 Fluconazole+ Caspofungin:1	2	0	NR
Wang et al. [64]	5	5	-	5	-	-	67	42	7.4	-	5	-	-	-	-	-	2 weeks ev 4–8 weeks os 2 weeks ev (after reimplantation)	Fluconazole + cefuroxime + levofloxacine ev (2 weeks) Fluconazolo + levofloxacine + riphampicin os (4–8 weeks) Fluconazole + cefuroxime + levofloxacine ev (2 weeks) — after reimplantation	0	0	-
Kim et al. [65]	9	9	-	9	-	-	76	66	NR	-	9	-	-	-	-	-	3 months: 2 4 months: 1 6 months: 1 12 months: 2 14 months: 1 15 months: 2	NR	0	0	-
Baecker et al. [72]	18	18	11	7	-	-	72	35	NR	-	-	18	-	-	-	Arthrodesis: 3 Girdlestone: 2	6 months	Fluconazole	0	0	-
George et al. [66]	2	2	-	2	-	-	60	33	60.5	2	-	-	-	-	-	-	4 months	Fluconazole	0	0	-
Mafrachi et al. [22]	1	1	-	1	-	-	60	12	9	-	1	-	-	-	-	-	15 months	Caspofungin + Fluconazol ev, Fluconazol os Fluconazol + amphotericin B ev Fluconazolo os (12 months)	0	0	-
Enz et al. [73]	145	18	14	4	-	-	70	NR	NR	-	7	-	1		Amputation: 9	-	NR	NR	3	0	NR
Brown et al. [74]	31	31	13	18	-	-	9	48	NR	1	21	-	5		-	Permanent spacer: 4	NR	Fluconazole	12	0	NR
Sidhu et al. [8]	1189	22	8	14	-	-	64	NR	36.4	-	22	-	-	-	-	Amputation: 4 Girdlestone: 1	1.5 months	Voriconazole: 19 Voriconazole + caspofungin 2 Voriconazole + Ambisone + Itraconazole: 1	8	6	NR
Ornell et al. [23]	1	1	-	-	-	1	57	3	1.5	-	1	-	-	-	-	Died: 1	6 months	Fluconazole	0	0	-
Kurmis et al. [24]	1	1	-	1	-	-	70	NR	12	-	1	-	-	-	-	-	NR	NR	0	0	-
Riaz et al. [75]	41	41	21	19	1	-	65	NR	NR	-	-	-	-	-	.	.	NR	NR	0	0	-
Gao et al. [76]	17	18	5	13	-	-	61	65	NR	-	18	-	-	-	-	5	NR	Fluconazole Voriconazole	1	0	NR
Bartash et al. [25]	1	1	1	-	-	-	54	19	1	-	1	-	-	-	-	-	3 months	Posaconazole	0	0	-
Hasan et al. [26]	1	1	1	-	-	-	45	3	36	-	-	-	-	1	-	-	NR	NR	0	0	-
Yilmaz et al. [27]	1	1	-	1	-	-	81	48	NR	-	1	-	-	-	-	-	6 weeks	Amphotericin B	0	0	-
Baumann et al. [28]	1	1	-	1	-	-	32	84	55	-	1	-	-	-	-	-	9 months	Amphotericin B + Fluconazole	0	0	-
Austin et al. [29]	1	1	-	1	-	-	80	NR	25	-	1	-	-	-	-	Arthrodesis	3 months	Amphotericin B	0	0	-
Cobo et al. [30]	1	1	1	-	-	-	77	5	48	-	-	-	-	1	-	-	6 months	Caspofungin + Fluconazole	0	0	-
Kelesidis et al. [31]	1	1	1	-	-	-	93	12	5	-	-	-	-	1	-	-	NR	Fluconazole	0	0	-
Graw et al. [67]	2	2	-	2	-	-	69	136	3	-	2	-	-	-	-	-	4 months	Voriconazole + Caspofungin Caspofungin + Fluconazole	0	0	-
Bland et al. [32]	1	1	-	1	-	-	55	NR	2	-	-	-	1	-	-	-	2 months	Fluconazol + micafungin	1	0	2
Gaston et al. [33]	1	1	-	1	-	-	42	NR	240	-	-	-	1	-	-	-	NR	Amphotericin B (endoarticular)	1	1	2
Phelan et al. [68]	4	4	3	1	-	-	74	NR	22	-	4	-	-	-	-	-	4-6 months	Amphotericin B + Fluconazole	0	0	-
Lackner et al. [34]	1	1	-	1	-	-	61	122	1	-	1	-	-	-	-	Amputation: 1	NR	Itroconazol + voriconazole	1	0	6
Johannssonet al. [35]	1	1	1	-	-	-	84	4	108	-	1	-	-	-	-	-	4 months	Aphootericin B + fluconazol	0	0	-
Gottesman-Yekutieli et al. [36]	1	1	1	-	-	-	56	12	24	-	1	-	-	-	-	-	10 months	Voriconazole	0	0	-
Fowler et al. [37]	1	1	1	-	-	-	84	36	156	-	-	-	1	-	-	-	NR	Itroconazole	0	0	-
Dutronc, et al. [69]	7	7	3	4	-	-	72	30	3.7	-	5	-	1	1	-	Arthrodesis: 1	55 months	NR	0	0	-
DeHart. et al. [38]	1	1	-	1	-	-	56	30	NR	-	-	-	-	1	-	-	30 months	Itroconazole + Amphotericin B	0	0	-
Deelstra et al. [39]	1	1	1	-	-	-	73	72	6	-	1	-	-	-	-	-	NR	Fluconazole	0	0	-
Azzam et al. [3]	31	31	14	17	-	-	64	45	25	-	29	-	2	-	-	Arthrodesis: 3 Amputation: 4	7.5 months	Fluconazole: 23 Amphotericin B: 5 Caspofungin: 3	10	5	NR
Austen et al. [40]	1	1	-	1	-	-	77	6	60	-	-	-	-	1	-	-	4 months	Fluconazole	0	0	-
Nayeri et al. [41]	1	1	1	-	-	-	62	24	60	1	-	-	-	-	-	-	NR	Flucitosine + Amphotericin B	0	0	-
Marra et al. [42]	1	1	1	-	-	-	59	NR	0.5	-	1	-	-	-	-	-	NR	Fluconazole	0	0	-
Açikgöz et al. [43]	1	1	-	1	-	-	70	5	9	-	1	-	-	-	-	Arthrodesis: 1	NR	Fluconazole	0	0	-
Reddy et al. [44]	1	1	-	1	-	-	62	24	24	-	1	-	-	-	-	-	4.5 months	Fluconazole	0	0	-
Zhu et al. [45]	1	1	1	-	-	-	44	3	1	-	-	-	1	-	-	-	1.5 months	Amphotericin B + Voriconazole	0	0	-
Fukasawa et al. [46]	1	1	-	1	-	-	80	24	1	-	-	-	1	-	-	-	1 month	Fluconazole	0	0	-
Wada et al. [47]	1	1	-	1	-	-	77	36	NR	-	-	-	1	-	-	-	NR	Fluconazole	0	0	-
Lazzarini et al. [48]	1	1	1	-	-	-	63	24	7	-	1	-	-	-	-	-	2 months	Amphotericin B	0	0	-
Artiaco et al. [49]	1	1	1	-	-	-	70	NR	60	-	-	-	-	1	-	-	6 months	Fluconazole	1	0	12
Jenny et al. [50]	2	2	2	-	-	-	65	30	42	2	-	-	-	-	-	-	2 months	Voriconazole + Flucitosine	0	0	-
Merrer et al. [12]	1	1	1	-	-	-	81	11	144	-	-	-	-	1	-	-	10 months	Fluconazole	0	0	-
Anagnostakos et al. [15]	7	7	4	3	-	-	68	28	NR	-	7	-	-	-	-	-	1.5 months	Fluconazole: 5 Voriconazole: 1 Caspofungin: 1	0	0	-
Paul et al. [51]	1	1	-	1	-	-	63	24	20	-	1	-	-	-	-	Arthrodesis: 1	2 months	Amphotericin B + Fluocitosine	0	0	-
Selmon et al. [52]	1	1	-	1	-	-	75	48	84	1	-	-	-	-	-	-	2 months	Itraconazole	0	0	-
Ramamohan et al. [53]	1	1	1	-	-	-	65	24	11	-	1	-	-	-	-	-	1.5 months	Amphotericin B + Flucitosine	0	0	-
Bruce et al. [54]	2	2	2	-	-	-	60	132	60	-	2	-	-	-	-	-	NR	Fluconazole	0	0	-
Lidder et al. [55]	1	1	1	-	-	-	76	24	24	-	1	-	-	-	-	-	6 months	Amphotericin B	0	0	-
Yang et al. [56]	1	1	-	1	-	-	68	48	24	-	1	-	-	-	-	-	1 month	Fluconazole	0	0	-
Wu et al. [14]	1	1	-	1	-	-	72	12	24	-	1	-	-	-	-	-	4 months	Fluconazole	0	0	-
Ueng et al. [11]	16	16	7	9	-	-	62	41	21	-	16	-	-	-	-	Persistent spacer	10 months	Fluconazole	1	0	18
Lichtman [57]	1	1	-	-	1	-	59	NR	30	-	1	-	-	-	-	-	NR	Amphotericin B + ketoconazole	0	0	-
Lambertus et al. [58]	2	2	1	1	-	-	63	NR	10	-	2	-	-	-	-	-	6 months	Amphotericin B + ketoconazole	0	0	-
Darouiche et al. [70]	10	10	5	4	1	-	63	16	11.5	-	10	-	-	-	-	Arthrodesis: 3	1 month	Amphotericin B	0	0	-
Tunkel et al. [59]	1	1	-	1	-	-	37	12	72	-	1	-	-	-	-	-	NR	Amphotericin B + ketoconazole	1	1	1
Cushing et al. [60]	1	1	-	1	-	-	73	12	30	-	-	-	-	1	-	-	12 months	Fluconazole	0	0	-
Simonian et al. [61]	1	1	-	1	-	-	79	72	36	-	-	-	-	1	-	-	8 months	Ketoconazole	0	0	-
Brooks et al. [62]	1	1	-	1	-	-	64	24	9	-	-	-	1	-	-	-	7 months	Amphotericin B + Fluconazole	0	0	-
Klatte et al. [71]	10	10	6	4	-	-	68	96	25	-	-	-	-	-	-	-	1.5 months	3 Flucytosin + Amphotericin B + Fluconazole 2 Voriconazole 4 Flucytosin + Amphotericin B 1 Fluconazole	1	0	NR
Ji et al. [80]	11	11	4	7	-	-	66	12	NR	11	-	-	-	-	-	-	4 months	9 Fluconazole 2 Voriconazole	4	0	15

PJI: prosthetic joint infection; DAI: debridement and implant retention; *: Bilateral PJI; THA: total hip arthroplasty; TKA: total knee arthroplasty; NR: not reported.

**Table 2:** Most frequently reported pathogens.

Pathogen	PJI, n	PJI, %
*C. Albicans*	172	49%
*C. Parapsilosis*	94	27%
*C. Glabrata*	21	6%
*C. Tropicalis*	11	3%
*A. Fumigatus*	9	3%
*Aspergillus* spp.	5	1%
*C. Guillermondii*	4	1%
*C. Famata*	4	1%

Among the isolated microorganisms, the most prevalent were *Candida* spp.: *C. albicans* were responsible for 49% of cases followed by *C. parapsilosis* (27%) and *C. glabrata* (6%). (Table 2) Other less common Candida species included *C. pelliculosa*, *C. lipolytica*, *C. utilis*, *C. dubliniensis*, and *C. freschussii*, each reported in a single case. Other reported fungi comprised *Aspergillus Fumigatus* (3%). In five additional cases, (1%) *Aspergillus* spp. were reported with no further details. In a small number of patients other fungi were identified as the pathogenic organism: *Pithomyces*, *Penicillium*, *Rhodotorula minuta*, *Virticillium*, *Blastoschizomyces capitatus*, *Alternaria*, *Trichosporon* and *Aureobasidium*, although this is not an exhaustive list. 

The use of many different antifungal agents has been reported. The choice was dependent on case-specific pathogens and medical comorbidities. The duration of intravenous therapy was also variable between studies with an average of 4 months, even if not reported in all studies. Intravenous regimens were typically followed by prolonged courses of oral antifungals. Fluconazole was the most used, both as a monotherapy and in combination. Intravenous amphotericin B was also used, often in combination with either fluconazole or flucytosine. The use of echinocandins (micafungin, anidulafungin, and caspofungin) was also described. 

A two-stage exchange was the primary surgical procedure in 239 patients. A one-stage approach and DAIR were reported in 30 (8.0%) and 18 (4.8%) cases, respectively, mainly as case reports. In a small case series reporting on 10 cases treated with a single-stage approach, Klatte et al. [71] observed only one case with a recurrence of infection out of 10 cases. On the other hand, Ji et al. [80] observed a 36% fPJI recurrence rate. All other data on DAIR and one-stage can only be deduced by summarizing sparse and heterogeneous data reported in the literature. Success rates of the two-stage approach also vary widely. Azzam et al. [3] reported a success rate of 47.4% among 31 cases. Phelan et al. [68] described an eradication rate of 80%. In addition, Anagnostakos et al. [15] observed no recurrence among seven cases of fPJI treated by the two-stage exchange protocol, and Ueng et al. [11] reported 16 patients with a success rate of 50%.

A three-stage approach with planned spacer exchange was reported by Baecker et al. [72] in 18 cases with a 72% rate of reimplantation.

## 4. Discussion

Only a few publications described the detection rate of fungi in PJIs in total, reporting a 0.9–2.0% rate [8,74,81,82,83]. However, Enz et al. observed [73] a much higher incidence of fPJI, detecting a fungus in 12.4% of the whole PJI cohort. 

The diagnosis of fPJI is challenging because of its chronic and indolent clinical course. Upon physical examination, patients frequently only manifest mild symptoms (pain, swelling, and/or reduced range of motion in the absence of acute local inflammatory signs) that are similar to those of low-grade PJI [84]. A sinus tract represents a major diagnostic criterium according to the 2018 Musculoskeletal Infection Society (MSIS) criteria [85]; however, it was only reported in 0% [3] to 16% of cases [74]. Fungal PJI should therefore be suspected in patients with one or more specific risk factors [8,63], such as immunosuppression, obesity, diabetes, many previous revision surgeries as well as long-term antibiotic treatments [3,11,17,18,30,63,74,75,77,78,79]. Moreover, chronic medical conditions such as prolonged steroid use, renal disease, malignancy, rheumatoid arthritis, chemotherapy, and hepatic diseases, which can contribute to poor host immunity, can increase the risk of fungal infections [65]. Riaz et al. [75] reported that antimicrobial therapy within three months before the diagnosis of PJI and the presence of wound drainage lasting longer than five days prior to the diagnosis of PJI is significantly associated with increased odds of fPJI when compared with bacterial PJI. Nonetheless, a recent study [30] reported that 32% of fPJI patients have no risk factors.

Systemic inflammatory markers, including erythrocyte sedimentation rate (ESR), C-reactive protein (CRP), and D-dimer, are the first screening tool for PJI, but they are generally minimally elevated or even normal [75,86,87]. In the presence of high clinical suspicion, synovial fluid should be sampled and sent for testing and culture [88]. 

Additionally, an underestimation of the prevalence of fPJI can be due to the difficulty in culturing fungi. To optimize the diagnostic process, the use of selective fungal media and an adequate incubation time of 5 to 14 days is recommended [89]. In some culture-negative cases, uncultivable organisms must be taken into account and alternative identification techniques must be considered, such as polymerase chain reaction (PCR) and next-generation sequencing (NGS) [6]. Despite no uniform diagnostic standards existing for fPJI, according to the European Bone and Joint Infection Society’s (EBJIS) definition of PJI [90], the isolation of an uncommon or highly virulent organism on only one sample should be considered a probable infection.

The significant variability of pathogens associated with fPJI further complicates their management. The most identified fungus is *Candida albicans* (in approximately 49% of cases) followed by *Candida parapsilosis* (27%) [75,91]. *Candida albicans* forms complex biofilms compared to other *Candida* spp. [91,92], thus contributing to its pathogenicity. Only 14 cases of fPJI caused by *Aspergillus* spp. have been reported in the literature [93]. Moreover, a high rate of mixed bacterial and fPJI have been reported (ranging between 16% [74] and 33% [17] of fPJIs), which increases difficulties in their diagnosis and treatment. Mixed infections are associated with significantly worse outcomes. Sidhu et al. [8] described a revision-free survival rate of 38% in mixed infections. Bacteria and fungi establish a mutually beneficial mixed biofilm that protects them from antimicrobial treatment [73]. For example, *Staphylococci* can affect the activity of antifungal drugs while staphylococcal proteinase can enhance the adhesion capability of *C. albicans* [94], thus favoring the survival of the yeast. On the other hand, the presence of Candida species may increase the growth of anaerobic bacteria by generating a hypoxic microenvironment [95], stimulate biofilm development in *Enterococcus faecalis* strains (which are normally unable to form biofilm on their own [96]), and lead to an enhanced tolerance of *Staphylococcus aureus* towards vancomycin [97].

Implant removal is of paramount importance for the successful treatment of fPJI [98]. A total of 18 cases treated with DAIR have been reported, often resulting in persistent infection requiring multiple revisions [5,12,32,33,46,61,74,80]. However, most of these data can be only deduced by extrapolating sparse data reported in the literature. Moreover, most of these cases are reported together with other treatments in heterogeneous series.

A one-stage approach was reported in 30 cases with discordant results. In a small case series reporting on 10 cases treated with a single-stage approach, Klatte et al. [71] observed only one case with a recurrence of infection out of 10 cases. On the other hand, Ji et al. [80] observed a 36% fPJI recurrence rate. One-stage exchange might be considered in unfit patients who are less likely to tolerate multiple procedures; however, similarly to bacteria PJI, the pathogen involved must be well studied, and the response to antifungal treatment must be predictable [71]. In the case of rare or resistant pathogens, a single-stage exchange is often ineffective. However, the interpretation of treatment success is difficult because of the small number and heterogeneity of the described cases [52,71,80]. 

Two-stage exchange is the most commonly used surgical approach for fPJI with rates of infection eradication up to above 90% [99]. However, success rates vary widely. Kuiper et al. [17] reviewed 164 cases of fPJI and reported a success rate for the two-stage exchange protocol of 84.8%. In the series by Azzam et al. [3], the success rate was 47.4%. Phelan et al. [68] reported a success rate of 80%. Anagnostakos et al. [15] described seven cases of fPJI treated by the two-stage exchange protocol without any recurrence. A therapy protocol with three-stage revision has also been described [72]. Repeated surgical debridement combined with a scheduled spacer exchange could facilitate continuous delivery of the highest local drug concentrations with optimized release kinetics [72,100]. 

The use of cement spacers loaded with antifungal agents has been suggested to be effective in eradicating local infections and reducing the duration of antifungal treatment. However, no definitive evidence exists on which type and dosage of drug need to be incorporated to achieve the best results [101]. Amphotericin is the most used, due to its relative heat stability in polymethylmethacrylate (PMMA) cement. However, Cunningham et al. [102] reported a decrease in compressive strength of the spacer over time with the addition of amphotericin. Voriconazole has shown some promise and is becoming increasingly used in cement spacers: it has been shown to have predictable elution in vitro and determines only a mild reduction in compressive strength [103].

In addition, there is no agreement on the choice of optimal medical treatment. The choice of antifungal agent should be driven by local patterns of resistance and patient factors [3,30]. Fluconazole and amphotericin B are agents characterized by good bone penetration and are effective against most fungi [17,78]. Unfortunately, they have several side effects, especially in patients with renal and hepatic impairment. Echinocandins, such as micafungin, caspofungin, and anidulafungin, have a more tolerable side effect profile [101]. There is also no single consensus regarding the duration of antifungal administration. In consideration of the indolent nature of fPJIs, the ideal interval between implant removal and reimplantation are unknown. For patients who are not candidates for surgery, lifelong suppressive therapy with oral antifungals, such as fluconazole, is mandatory [3,104]. For Candida infections, the European Society for Clinical Microbiology and Infectious Disease recommends implant removal with at least 14 days of parenteral antifungals followed by a subsequent minimum of 4 to 6 weeks of oral agents [105,106]. In the case of two-stage exchange, the International Consensus Meeting (ICM) recommends a minimum of 6 weeks treatment after prosthesis removal [107]. The Infectious Diseases Society of America (IDSA) guidelines recommend between 6 and 12 months [10]. A meta-analysis by Ueng et al. [11]. identified improved eradication of infection with prolonged systemic therapy from three to six months. However, Anagnostakos et al. [15] suggested that 6 weeks of administration of an antifungal agent can be sufficient. Reduced susceptibility of fungal organisms to antifungal agents has become an area of interest, with resistance being described in fPJI [52,108], particularly azole resistance in Candida PJI [67,109].

There are several limitations to this study. Many of the included studies are case reports or small case series. There is a real lack of long-term data on fungal PJI, with many series not reporting the outcome [17,110,111]. Additionally, many series used different outcome measurements. Moreover, the heterogeneity of reported pathogens does not allow for the determination of the correlation between pathogen type and treatment outcomes.

In conclusion, fungal PJI is a rare complication compared to bacteria PJI. The present literature review highlights the extreme heterogeneity in the reported treatments of fungal prosthetic joint infections. In particular, the most conspicuous discrepancies emerge in medical management both in terms of drug of choice and length of treatments. A two-stage revision arthroplasty is generally suggested for fPJI, considering it usually presents as delayed or late infection. Nonetheless, success rates were less favorable than in bacterial PJI, and patients affected by fPJI treated with a two-stage exchange protocol were more likely to relapse compared with patients with bacterial PJI that underwent the same treatment. The need for multicenter, prospective studies to provide standardized protocols and to improve the treatment of fungal PJI clearly emerges.

## Data Availability

The data presented in this study are available on request from the corresponding author.
